# Retraction: The two-component system CpxR/A represses the expression of *Salmonella* virulence genes by affecting the stability of the transcriptional regulator HilD

**DOI:** 10.3389/fmicb.2025.1568769

**Published:** 2025-02-03

**Authors:** Frontiers Editorial Office

The journal retracts the August 6 2015 article cited above.

Following publication, concerns were raised by the authors regarding image manipulation in the published figures. The submitting author, Miguel Ángel De la Cruz, has acknowledged and accepted responsibility for inappropriate manipulation of the manuscript figures.

Image duplication and manipulation was confirmed in Figures 1C, 2, 3A, B, 5B of the manuscript. As a result, the data and conclusions of the article have been deemed unreliable and the article has been retracted.

This retraction was approved by the Chief Editors of Frontiers in Microbiology and the Chief Executive Editor of Frontiers. The authors agree to the retraction.

